# Unexpected Acute Pancreatitis After Tarlatamab in Small Cell Lung Cancer: A Case Report

**DOI:** 10.7759/cureus.117559

**Published:** 2026-10-06

**Authors:** Afoma Anyadibe, Saman Amjad, Eduzor Onyechi, Anuoluwa Oyetoran, Jessica Thomas, Waqqas Tai, Ishmael Jaiyesimi

**Affiliations:** 1 Hematology and Oncology, Corewell Health William Beaumont University Hospital, Royal Oak, USA; 2 Internal Medicine, Corewell Health William Beaumont University Hospital, Royal Oak, USA; 3 Internal Medicine, SSM Health St. Mary's Hospital, Richmond Heights, USA

**Keywords:** acute pancreatitis, bispecific t-cell engager, immune-related adverse events, small cell lung cancer, tarlatamab

## Abstract

Tarlatamab is a bispecific T-cell engager (BiTE) targeting delta-like ligand 3, recently approved for relapsed extensive-stage small cell lung cancer. Its toxicity profile is primarily characterized by cytokine release syndrome and neurotoxicity. Pancreatitis has not been well described. We report a case of acute pancreatitis occurring approximately eight hours after the first dose, followed by completion of subsequent scheduled doses without recurrence. A 62-year-old man developed severe epigastric pain on cycle 1, day 1, after receiving tarlatamab. Lipase was elevated, and imaging confirmed acute interstitial pancreatitis. There was no history of alcohol use, and evaluation did not demonstrate gallstones, biliary obstruction, hypertriglyceridemia, hypercalcemia, or autoimmune disease. The patient improved with supportive care. After recovery, he proceeded with the remaining scheduled doses of cycle 1, which were completed without recurrence of symptoms. This case highlights a possible early, transient pancreatic toxicity and suggests that treatment may be completed in selected patients after resolution of symptoms.

## Introduction

Extensive-stage small cell lung cancer (ES-SCLC) has a poor prognosis, which has led to the development of novel therapies for relapsed disease. Tarlatamab (Imdelltra) is a bispecific T-cell engager (BiTE) antibody that binds delta-like ligand 3 on tumor cells and CD3 on T lymphocytes [1]. In the phase III DeLLphi-304 trial, tarlatamab demonstrated improved survival outcomes compared with chemotherapy in previously treated SCLC [2], leading to its approval as the first BiTE therapy for a solid tumor [3]. As an expected consequence of T-cell activation, commonly reported adverse effects include cytokine release syndrome and neurotoxicity [1].

Pancreatitis is not a commonly recognized adverse effect of tarlatamab. Across immunotherapy platforms, clinically apparent pancreatitis remains uncommon, occurring in well under 1% of patients receiving checkpoint inhibitors [4]. Only isolated cases have been described with other T-cell engager therapies, such as blinatumomab [5], and pancreatitis has been reported rarely in clinical trial data for tarlatamab [6]. We present a case of acute pancreatitis temporally associated with tarlatamab administration.

## Case presentation

A 62-year-old man with ES-SCLC and coexisting lung adenocarcinoma was referred for further treatment following radiographic and symptomatic progression, including new brain metastases. He had previously received carboplatin, etoposide, and atezolizumab, achieving a partial response. After progression, he was initiated on tarlatamab (Imdelltra) under supervised conditions.

On cycle 1, day 1, he received a 1-mg step-up dose of tarlatamab with dexamethasone premedication and antimicrobial prophylaxis (acyclovir and trimethoprim-sulfamethoxazole). He was admitted for routine observation. Approximately eight hours after completion of the infusion, he developed sudden, severe epigastric pain, rated 10/10, radiating to the back and associated with nausea.

On examination, he was afebrile and hemodynamically stable. Abdominal examination revealed marked epigastric tenderness without guarding or rebound. Laboratory studies demonstrated leukocytosis with a left shift. Hemoglobin and platelet counts were within normal limits. Liver function tests, serum calcium, triglycerides, and IgG4 levels were within reference ranges. Serum lipase was elevated. There was no prior history of pancreatitis. Relevant laboratory values are summarized in Table 1.

**Table 1:** Laboratory findings on presentation. Reference ranges are institution-specific.

Laboratory test	Result	Reference range
White blood cell count	12.4 × 10⁹/L	3.5-10.1 × 10⁹/L
Serum lipase	781 U/L	7-60 U/L
Triglycerides	107 mg/dL	<150 mg/dL

CT of the abdomen and pelvis with IV contrast demonstrated peripancreatic inflammatory changes and mild fluid surrounding the pancreatic tail, consistent with acute interstitial pancreatitis (Figure 1). 

**Figure 1:**
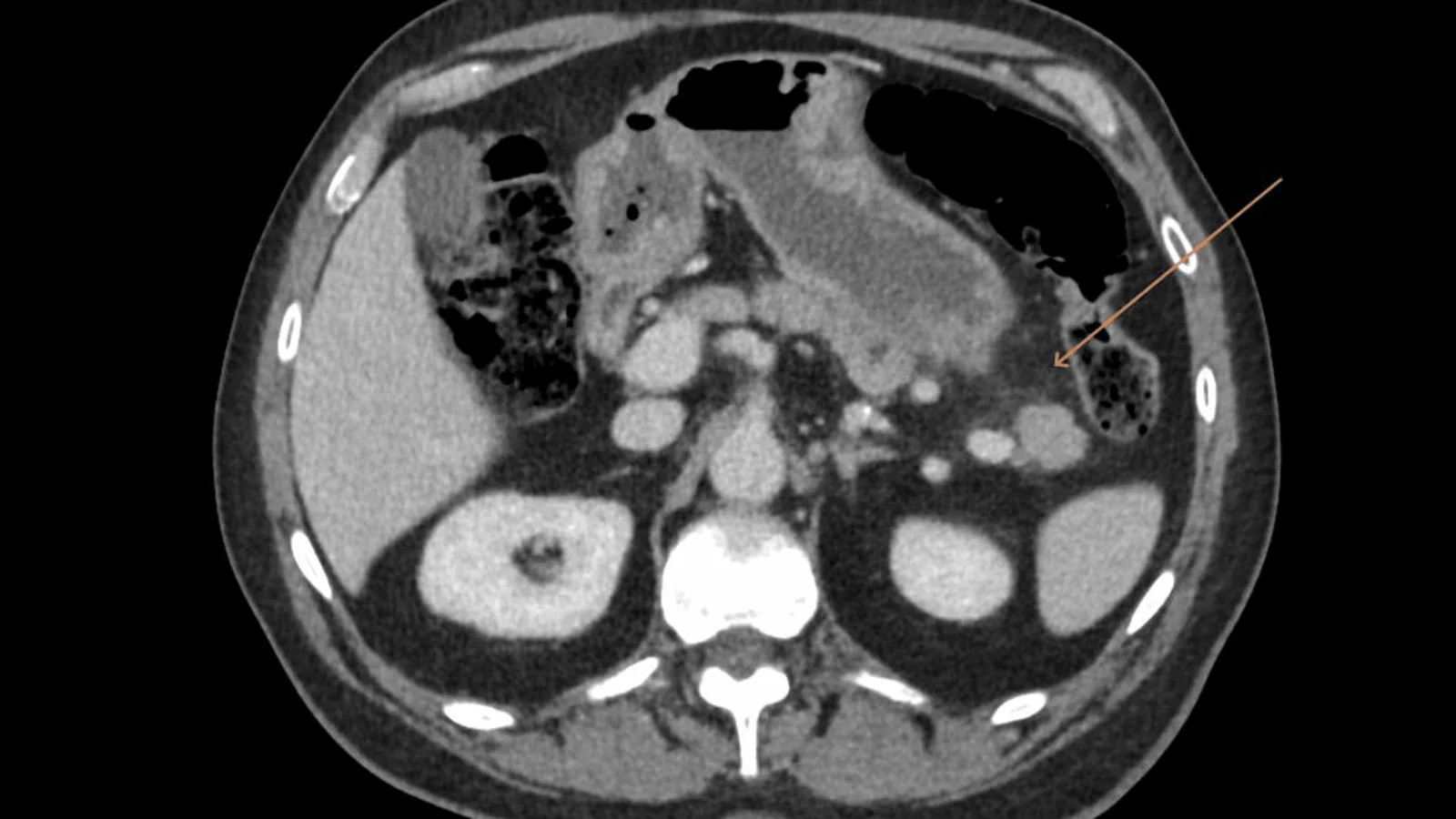
Axial CT abdomen demonstrating peripancreatic inflammation. Axial contrast-enhanced CT image of the abdomen demonstrating peripancreatic fat stranding and inflammatory changes surrounding the pancreatic tail (arrow), consistent with acute interstitial pancreatitis.

There was no evidence of gallstones, biliary dilation, or pancreatic mass. Right upper quadrant ultrasound demonstrated no cholelithiasis or intrahepatic or extrahepatic biliary ductal dilation, and the common bile duct measured 5 mm. The patient denied any history of alcohol use.

He was managed conservatively with IV fluids, bowel rest, and analgesia. Steroids were continued for the management of brain metastases. His abdominal pain improved over three to four days, and he resumed oral intake. Lipase levels declined in parallel with symptom resolution (Figure 2).

**Figure 2:**
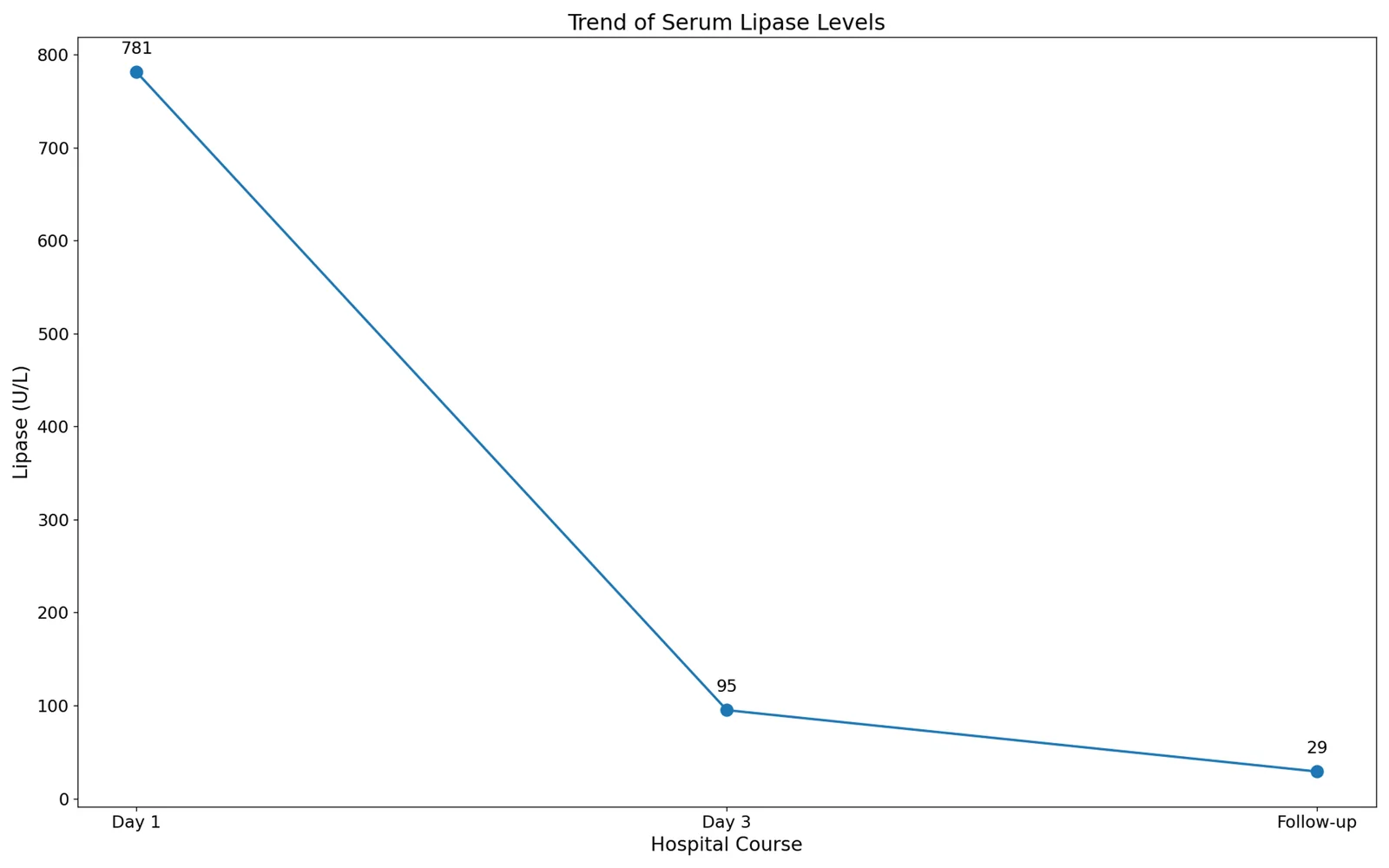
Trend of serum lipase levels during hospital course. Graph demonstrating serum lipase levels peaking on hospital day 1 (781 U/L), with a subsequent decline by day 3 and normalization at outpatient follow-up, consistent with clinical improvement following supportive management.

Given the close temporal relationship with tarlatamab administration and the absence of an alternative etiology, a possible drug-related association was considered. After clinical recovery, the patient proceeded with the remaining scheduled doses of cycle 1 (days 8 and 15), which were completed without recurrence of abdominal pain or elevation in pancreatic enzymes.

## Discussion

Tarlatamab is a BiTE that induces T-cell activation and cytokine release, a mechanism that underlies both its therapeutic effect and associated toxicities [1,3]. This immune activation is central to its antitumor efficacy but may also predispose patients to inflammatory adverse events affecting multiple organ systems. Acute pancreatitis is an extremely rare adverse event in patients receiving tarlatamab. In available clinical trial data, only a single case (approximately 0.5%) has been reported [6], and detailed clinical descriptions remain limited. As use of this agent expands in clinical practice, recognition of less common toxicities becomes increasingly important. This case adds to the emerging safety profile and provides additional clinical context regarding presentation and management.

The clinical presentation in this case supports a likely treatment-related event. Symptoms developed approximately eight hours after the first dose, and common etiologies were systematically excluded. There was no history of alcohol use, no evidence of gallstones or biliary obstruction on imaging, and laboratory evaluation demonstrated normal triglyceride and calcium levels. Autoimmune pancreatitis was unlikely given normal IgG4 levels. The timing of symptom onset in close proximity to drug administration further supports a causal association, although definitive attribution remains challenging in the absence of specific biomarkers. In addition, pancreatitis has been reported, although infrequently, with immune checkpoint inhibitors [4]. Blinatumomab, another T-cell-engaging therapy, has also been associated with rare cases of pancreatitis, likely mediated by cytokine-driven inflammation [5]. Although pancreatic metastasis from small cell lung cancer has been reported as a rare cause of pancreatitis, no such findings were identified on imaging in this case [7]. Together, these observations support the concept that pancreatic injury may occur as part of a broader spectrum of immune-related toxicities.

Cytokine-mediated inflammatory pathways likely play a central role in the pathogenesis. Elevations in interleukin-6, tumor necrosis factor-α, and interferon-γ may contribute to pancreatic injury through endothelial dysfunction, increased vascular permeability, and localized hypoperfusion [8]. These mechanisms have been described in the context of cytokine release syndromes and may extend to pancreatic tissue in susceptible individuals. Further data from systematic reviews and clinical series support the occurrence of pancreatic injury in patients receiving immune-based therapies, although the overall incidence remains low [9,10].

An important aspect of this case is the absence of recurrence following completion of subsequent scheduled doses after recovery. This suggests that the event may have been transient or idiosyncratic rather than a reproducible toxicity. While data on continuation or rechallenge after suspected immune-related pancreatitis are limited, this experience suggests that, in carefully selected patients with complete resolution of symptoms and normalization of laboratory parameters, treatment may be resumed with close monitoring.

From a clinical standpoint, new-onset abdominal pain in patients receiving T-cell engager therapy should prompt early evaluation for pancreatitis, including laboratory assessment and imaging when appropriate. Early recognition allows for prompt supportive management and may prevent complications. At the same time, this case suggests that a single episode does not necessarily preclude completion of therapy when alternative causes have been excluded and the clinical course is favorable. Further reporting of similar cases will be important to better define the incidence, underlying mechanisms, and optimal management strategies for pancreatic toxicity associated with T-cell engager therapies. As these agents become more widely used, accumulating real-world data will be essential to guide clinical decision-making and risk stratification.

## Conclusions

We report a case of acute pancreatitis temporally associated with tarlatamab in a patient with ES-SCLC. After exclusion of common etiologies, the presentation was considered possibly treatment-related. The pancreatitis resolved with supportive care, highlighting the importance of early recognition of potential immune-related toxicities. Notably, the patient was able to complete subsequent scheduled doses after recovery without recurrence; however, decisions regarding rechallenge should be individualized. Clinicians should consider pancreatitis in patients receiving T-cell engager therapy who present with acute abdominal pain.
